# Preparing for future waves and pandemics: a global hospital survey on infection control measures and infection rates in COVID-19

**DOI:** 10.1186/s13756-021-01029-z

**Published:** 2021-12-20

**Authors:** Simon Matoori, Daniel R. Kuritzkes, Yonggeng Goh, Swee Tian Quek, Liang Wang, Ziyan Sun, Fabiano di Marco, Daniela Borleri, Sabrina Buoro, Stefano Fagiuoli, Tatiana Ferrari, Marco Rizzi, Federico Raimondi, Simonetta Cesa, Dow-Mu Koh, Johannes M. Froehlich, Sonja Janssen, Bettina Lange, Alexander Egle, Stefan Erb, Erik Mossdorf, Andreas Gutzeit

**Affiliations:** 1 Faculté de Pharmacie, Université de Montréal, 2940 Chemin de Polytechnique, Montreal, QC H3T 1J4 Canada; 2 Department of Health Sciences and Medicine, University of Lucerne, Lucerne, Switzerland; 3 Department of Radiology, Paracelsus Medical University, Salzburg, Austria; 4 Division of Infectious Diseases, Brigham and Women’s Hospital, Boston, MA USA; 5 Department of Diagnostic Imaging, National University Hospital, Singapore, Republic of Singapore; 6 Department of Radiology, Tongji Hospital, Tongji Medical College, Huazhong University of Science and Technology, Wuhan, China; 7 Pneumology Unit, Papa Giovanni XXIII Hospital, Bergamo, Italy; 8 Health Sciences Department, University of Milan, Milan, Italy; 9 Occupational Medicine Unit, Papa Giovanni XXIII Hospital, Bergamo, Italy; 10 Quality Management Unit, Papa Giovanni XXIII Hospital, Bergamo, Italy; 11 Gastroenterology, Hepatology and Liver Transplantation, Department of Medicine, Papa Giovanni XXIII Hospital, Bergamo, Italy; 12 Infectious Diseases Unit, Papa Giovanni XXIII Hospital, Bergamo, Italy; 13 Respiratory Unit, Department of Medicine, Papa Giovanni XXIII Hospital, Bergamo, Italy; 14 Department of Health and Social Care Professions, Papa Giovanni XXIII Hospital, Bergamo, Italy; 15 Clinical Research Group, Klus Apotheke Zurich, Zurich, Switzerland; 16 Clinic of Radiology and Nuclear Medicine, University Medical Center Mannheim, University of Heidelberg, Mannheim, Germany; 17 Hospital Hygiene Unit, University Medical Center Mannheim, University of Heidelberg, Mannheim, Germany; 18 IIIrd Medical Department with Hematology and Medical Oncology, Hemostaseology, Rheumatology and Infectious Diseases, Oncologic Center, Paracelsus Medical University, Salzburg, Austria; 19 Department of Infectious Diseases, Hirslanden Klinik St. Anna, Lucerne, Switzerland

## Abstract

A survey of hospitals on three continents was performed to assess their infection control preparedness and measures, and their infection rate in hospital health care workers during the COVID-19 pandemic. All surveyed hospitals used similar PPE but differences in preparedness, PPE shortages, and infection rates were reported.

**Supplementary Information:**

The online version contains supplementary material available at 10.1186/s13756-021-01029-z.

## Introduction

In the COVID-19 pandemic, the need for personal protective equipment (PPE) strongly increased to protect health care workers (HCWs) within the hospital from infection by COVID-19-positive patients [1]. Although our understanding of the novel coronavirus strain SARS-CoV-2 has been rapidly evolving, a detailed understanding on the modes of transmission is still lacking [2, 3]. To protect HCWs, the World Health Organization and the Centers for Disease Control recommend the use of face masks, eye protection, gowns, medical gloves, frequent hand hygiene, environmental cleaning, and waste management [4]. The most potent protection, respirators which filter out at least 94% (FFP2, N95) or 99% (FFP3, N99) of airborne particles, as well as other PPE were quickly in short demand as the pandemic swept around the planet, resulting in shortages in many hospitals with a potential increase in COVID-19 infection risk for HCWs [5, 6].

Our hypothesis was that the preparedness and response to the pandemic was different among hospitals, countries, and regions, and resulted in different exposure risk for HCWs in terms of COVID-19 prevalence and PPE availability. The goal of our study was to compare and assess the preparedness, the response, and the infection rate among hospital HCWs of several institutions on three continents by questionnaire.

## Methods

No Institutional Review Board approval or informed patient consent was needed for this survey. The survey was performed between June and August 2020 at eight different institutions: Salzburg (Austria), Wuhan (China), Mannheim (Germany), Bergamo (Italy), Singapore, Hirslanden Klinik in Lucerne (Switzerland), an anonymized institution in the UK, Boston (MA, USA). Sites were recruited by emailing the Division of Infectious Diseases of each hospital. Surveys were sent out by email and reports were received by email.

### Questionnaire

The surveyed hospitals were given a questionnaire about institutional baseline characteristics and infection control measures for the COVID-19 pandemic of 2020. The questionnaire was answered by an infectious disease expert of the corresponding institution, and included the following topics: (1) Baseline characteristics of the participating hospitals; (2) COVID-19 infection rates among HCWs, (3) Pandemic preparations and trainings for a pandemic with an infectious respiratory disease before the COVID-19 outbreak, (4) Personal protective equipment (PPE) for health care worker with close contact with COVID-19 positive or suspected patients during the COVID-19 pandemic, and (5) various management issues of the COVID-19 pandemic (Additional file 1).

The infection rate was calculated as the number of infected HCWs divided by the total number of HCWs in the hospital. Shortages in PPE were defined as the absence of the PPE article in question. Survey respondents retrieved all hospital infection data reported in this study from their hospital records.

## Results

### Baseline characteristics of the surveyed hospitals and COVID-19 infection rates

The overall survey response rate was 100%. No incomplete survey responses were received.

Baseline characteristics of the eight hospitals included in the survey and the COVID-19 case load of their country are shown in Table 1 and Additional file 1: Table S1. Five hospitals are located in Europe (Austria, Germany, Italy, Switzerland, United Kingdom), two in Asia (China, Singapore) and one in North America (United States) (Additional file 1: Fig. S1).

**Table 1:** Hospital baseline information and number of infected HCWs and patients

Institution	Total hospital beds	Total HCWs	Total infected HCWs	Percentage of infected HCWs (%)	Number of treated COVID-19 patients	Date of questionnaire completion
Salzburg, Austria	1167	3471	43	1.2	170	June 5, 2020
Wuhan, China	7000	9700	100	1.0	3200	July 23, 2020
Mannheim, Germany	1300	3719	17	0.4	63	June 3, 2020
Bergamo, Italy	1000	4705	398	8.5	2141^a^	September 24, 2020
Singapore	1160	7800	7	0.1	887	June 2, 2020
Lucerne, Switzerland	220	1300	6	0.4	18	August 10, 2020
UK	269	3900	278	7.1	95	August 1, 2020
Boston, USA	793	24,000	450	1.9	682^b^	August 18, 2020

^a^Number of hospital-admitted COVID-19 patients (total number in Emergency Department: 4212)

^b^Until June 30, 2020

All surveyed hospitals are mid-sized to large tertiary referral centers. The percentage of COVID-19-positive HCWs varied widely from 0.1 to 8.5%. The two Asian institutions showed low infection rates (≤ 1%) despite treating large numbers of COVID-19 positive patients.

### Pandemic preparedness before the COVID-19 outbreak

All hospitals except two (Italy and United Kingdom) reported respiratory infectious disease outbreak preparedness with basic infection control trainings of any kind before the onset of the COVID-19 pandemic (Additional file 1: Table S2).

The institutions in China and Singapore noted that their plan for a pandemic with a respiratory infection was mainly based on the experience from the first SARS epidemic and the resulting policies. While the institution in Italy did not specifically prepare for a pandemic with a respiratory infectious disease, there was a course to prepare for an Ebola outbreak aimed at the Emergency Department in 2019. Furthermore, there was an emergency preparation meeting before the first COVID-19 patient was admitted to the Italian institution.

### Personal protective equipment during the COVID-19 outbreak in case of close contact with COVID-19 patients

All surveyed hospitals reported the use of surgical masks or respirators (FFP2/3, N95) and other disposable PPE such as gloves, gowns, and hand disinfectants (Table 2). Disposable caps and goggles were not used in two European hospitals. Other PPE used include face shields, visors, aprons, and air-purifying respirators in aerosolizing procedures. HCWs were trained to put on and use the PPE in all institutions.

**Table 2:** Availability of PPE for frontline HCWs during the COVID-19 pandemic

Institution	Masks		Disposable gloves	Disposable gowns	Disposable caps	Disposable goggles	Hand disinfectant	Other PPE	Training
	Type	Duration							
Salzburg, Austria	SM, FFP2, FFP3	4 h	Yes	Yes	Yes	Yes^a^	Yes	Face shields	Yes
Wuhan, China	SM, N95, KN95	4 h	Yes	Yes	Yes	Yes	Yes	Yes	Yes
Mannheim, Germany	FFP2, FFP3	1/shift	Yes	Yes	Yes	Yes	Yes	Aprons	Yes
Bergamo, Italy	SM, FFP2, FFP3, N95, N99, KN95	1–2/shift	Yes	Yes	Yes	Yes	Yes	Face shields	Yes
Singapore	SM, N95	4–5 h	Yes	Yes	Yes	Yes	Yes	Powered air-purifying respirator (interventional radiology)	Yes
Lucerne, Switzerland	SM, FFP2	1/shift	Yes	Yes	No	Yes	Yes	No	Yes^b^
UK	SM, FFP2, FFP3	1/shift	Yes	Yes	Yes	No	Yes	Face shields	Yes^c^
Boston, USA	N95 (fitted)	1/shift (8–12 h)	Yes	Yes	No	Yes^a^	Yes	Personal respirators (if unable to use N95), face shields^a^	Yes

*SM* surgical masks

^a^Reused after disinfection

^b^Only HCWs in risk areas (COVID-19 ward, emergency department, intensive care unit)

^c^Only frontline staff with contact with COVID-19 patients

Four of the eight surveyed institutions reported shortages in PPE at some point in the pandemic (Additional file 1: Table S3). Masks or respirators were missing for at least a brief period of time at the institutions in Austria, Germany, Italy, and the US. Three of the eight institutions reported that disposable gowns were not available during the entire time of the pandemic. Single institutions further reported shortages in gloves and disinfectants, and the need for rationing PPE to avoid shortages.

### Management of the pandemic

Infectious disease specialists were in charge of the management of the pandemic in all surveyed hospitals except for the one in the UK where non-specialized physicians were responsible (Additional file 1: Table S4). All hospitals put a task force in charge of coordinating the safety measures to reduce viral spread in the hospital, and implemented zoning (i.e., areas designated for suspected and confirmed COVID-19 patients), grouping COVID-19-positive patients (cohorting), hygiene trainings, and restrictions for visitors. Masking of HCWs in the entire hospital was mandated by all hospitals except for the one in Salzburg, where it was recommended. Visitor restrictions were put in place in all surveyed hospitals, with two hospitals allowing no visitors and two only allowing compassionate visits.

## Discussion

In this study, we evaluated the preparedness, the management, and the infection rate of hospital HCWs of the COVID-19 pandemic of eight hospitals located in Europe, Asia, and North America. While most hospitals were prepared for a pandemic with a respiratory disease, there were shortages in several PPE, namely masks and gowns. The percentage of COVID-19-positive hospital workers ranged from 0.1 to 8.5%. Notably, the Asian hospitals reported no shortages in surgical masks or respirators and showed low infection rates (≤ 1%) despite high numbers of treated COVID-19 patients. Without pointing fingers, this survey helps us identify key aspects of preparedness in terms of planification and PPE item stockage, in order to be better prepared for future waves of COVID-19 and other pandemics with respiratory viruses.

The low infection numbers despite high caseloads in Asian hospital may be explained by the experience with the SARS epidemic in 2002/2003 which mainly affected Asian countries such as China, Singapore, Vietnam, and Hong Kong. Hospital transmission accounted for a 22 to 57% of SARS cases [7], and was primarily related to inappropriate or missing PPE, hand disinfection, and hospital personnel education (e.g., removal of contaminated PPE) [8]. As a result, these countries prepared sophisticated plans to manage future pandemics, mandating training hospital personnel for pandemics with respiratory pathogens, stockpiling PPE, and further measures such as zoning [9]. As no shortages in masks and respirators were reported by these hospitals, their stockpiling has likely contributed to the low infection rates in these hospitals, which underlines their importance in protecting HCWs from COVID-19 patients.

In our survey, countries with less exposure to the first SARS epidemic, namely in Europe and the US, were less prepared in terms of personnel training and/or PPE stockpiling, and experienced more PPE shortages and higher numbers of infected hospital HCWs. However, hospitals showed a steep learning curve, adopting measures such as universal masking for hospital personnel and proving their usefulness for infection control [10–12].

As we prepare for the next waves of COVID-19, we have learned that universal masking of hospital personnel with surgical masks, immediately isolating COVID-19-positive patients, and the use of highly protective PPE (respirators, eye protection, gowns) in contact with COVID-19 patients is of high utility to protect HCWs [4, 13]. As some of these insights were already available after the SARS epidemic and improved the response of the surveyed Asian hospitals to the COVID-19 pandemic, it is instrumental for hospitals to learn from each other’s experiences and to cooperate to improve hospital infection control in future waves of COVID-19 and other pandemics. Based on the information of this survey, it will be essential to either stock higher amounts of PPE or to have the capacity to produce or recycle PPE locally to be prepared for future disruptions in supply chains.

This study has several limitations. Most importantly, we cannot determine if the COVID-19 positive hospital HCWs were infected in the hospital or outside. Indeed, the true extent of COVID-19 is difficult to assess [14, 15]. Another limitation of the study is that the questionnaire was filled out when some countries were still in the first wave while others were past the first wave (e.g., China). Furthermore, there might be a selection bias as most hospitals were selected based on previous collaborations. Another limitation is that all surveyed hospitals are located in upper-middle to high-income countries. Finally, some surveyed European hospitals treated low numbers of COVID-19 patients, complicating the evaluation of their true preparedness.

In this survey, all responding hospitals in Asia, Europe, and North America reported the use of recommended PPE but the degree of preparedness, PPE shortages, and infection rate among hospital HCWs varied widely. Our study highlights the importance of universal masking of HCWs with surgical masks, immediately isolating COVID-19-positive patients, and the use of highly protective PPE (respirators, eye protection, gowns). To prepare for the next waves of COVID-19 and future pandemics, we further urge hospitals to learn from each other and to cooperate to improve infection control.

## Supplementary Information

**Additional file 1**: Supplementary Figure and Tables.

## Data Availability

All relevant data and material are included in the main text and the supporting information of this manuscript.
