# Supplementary material for: Preparing for future waves and pandemics: a global hospital survey on infection control measures and infection rates in COVID-19
Source: Antimicrob Resist Infect Control. 2021 Dec 20;10:170. doi: 10.1186/s13756-021-01029-z (PMC8685805; doi:10.1186/s13756-021-01029-z)
Supplement: Supplementary file 1 — Additional file 1: Supplementary Figure and Tables. [file 13756_2021_1029_MOESM1_ESM.docx]

**Preparing for future waves and pandemics: a global hospital survey on infection control measures and infection rates in COVID-19**

**Supplemental Material**


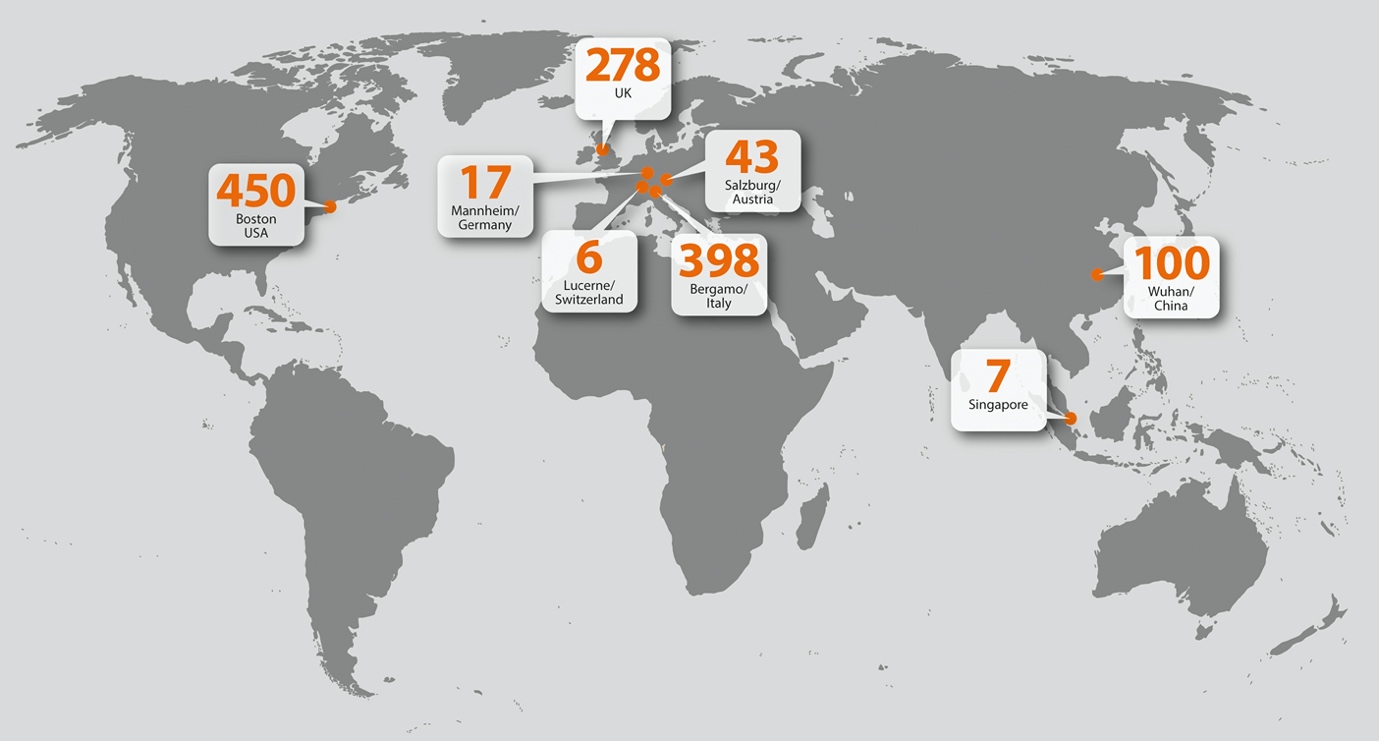
Supplementary Figure 1. Graphic representation of surveyed hospitals and infected HCWs.

Supplementary Table 1. COVID-19 case numbers in surveyed countries at the time of survey completion.

| **Country** | **Date of questionnaire completion** | **Cumulative COVID-19 case load on date of questionnaire completion**^1^ | **Population size in million (2020)**^2^ | **Normalized cumulative case load COVID-19**^3^  **(%)** |
| --- | --- | --- | --- | --- |
| Austria | June 5, 2020 | 16843 | 9.0 | 0.187 |
| China | July 23, 2020 | 83796 | 1439.3 | 0.006 |
| Germany | June 3, 2020 | 184121 | 83.8 | 0.220 |
| Italy | September 24, 2020 | 304323 | 60.5 | 0.503 |
| Singapore | June 2, 2020 | 35836 | 5.9 | 0.613 |
| Switzerland | August 10, 2020 | 36708 | 8.7 | 0.424 |
| UK | August 1, 2020 | 305562 | 67.9 | 0.450 |
| USA | August 18, 2020 | 5484630 | 331.0 | 1.657 |

^1^ Source: Our world in data (ourworldindata.com); ^2^ Source: Worldometer (worldometers.info); ^3^ Cumulative COVID-19 cases at time of questionnaire submission divided by total population of country in 2020

Supplementary Table 2. Preparations and trainings for a pandemic with an infectious respiratory disease before the COVID-19 outbreak.

| **Institution** | **Preparation and training** |
| --- | --- |
| Salzburg, Austria | Yes |
| Wuhan, China | Yes |
| Mannheim, Germany | Yes |
| Bergamo, Italy | No |
| Singapore | Yes |
| Lucerne, Switzerland | Yes |
| UK | No |
| Boston, USA | Yes |

Supplementary Table 3. Shortages in PPE during the COVID-19 pandemic.

| **Institution** | **Shortages** |
| --- | --- |
| Salzburg, Austria | Shortly in beginning of pandemic: masks such that the PPE standards had to be modelled on NHS and CDC emergency standards |
| Wuhan, China | Early stages: gowns |
| Mannheim, Germany | FFP2, FFP3, hand and surface disinfectants |
| Bergamo, Italy | Masks, gloves, gowns |
| Singapore | No |
| Lucerne, Switzerland | No |
| UK | No, but rationing |
| Boston, USA | Masks (particularly N95), gowns |

Supplementary Table 4. Management of pandemic in surveyed hospitals.

| **Institution** | **IDS** | | **Task force** | **Zoning** | **Cohorting >1 COVID-19+ per room** | **Whole hospital masking for HCW** | **Hygiene trainings** | **Restrictions for visitors** |
| --- | --- | --- | --- | --- | --- | --- | --- | --- |
|  | **Responsible** | **Number** |  |  |  |  |  |  |
| Salzburg, Austria | Yes | 3 | Yes | Yes | Yes | Recommended | Yes | Yes^3^ |
| Wuhan, China | Yes | 300 | Yes | Yes | Yes | Yes | Yes | Yes |
| Mannheim, Germany | Yes | 7 | Yes | Yes | No^2^ | Yes | Yes | Yes^3^ |
| Bergamo, Italy | Yes | 21 | Yes | Yes | Yes | Yes | Yes | Yes^4^ |
| Singapore | Yes | 20 | Yes | Yes | No^2^ | Yes | Yes | Yes^3^ |
| Lucerne, Switzerland | Yes | 1.5 | Yes | Yes | Yes | Yes | Yes | Yes |
| UK | No | - | Yes | Yes | Yes | Yes | Yes | Yes^4^ |
| Boston, USA | No^1^ | 50 | Yes | Yes | Rarely | Yes | Yes | Yes^3^ |

^1^Managed by infection control and occupational health; ^2^Isolation in single room; ^3^Only visits for terminal/palliative patients/compassionate reasons; ^4^No visitors allowed; IDS: infectious disease specialists.

**Questionnaire**

English version

1. Preparations **before** the COVID-19 outbreak

1.a) Were there preparations and trainings for a pandemic with an infectious respiratory disease before the COVID-19 outbreak?

2. Personal protective equipment **during the COVID-19 outbreak in case of close contact with COVID-19 patients**

2.a) Which type of masks/respirators were used during the COVID-19 outbreak: FFP2, FFP3, N95, KN95, or surgical masks? Other masks (e.g. fabric masks)? No masks?

2.b) Were disposable gloves used?

2.c) Were disposable gowns used?

2.d) Were disposable caps used?

2.e) Were disposable goggles used

2.f) Were other/additional protective material used?

2.g) Were hand disinfectants available?

2.h) Was there any shortage of protective material or disinfectants? If yes, which material? (Masks? Gloves? Gowns? Caps? Goggles? Disinfectants?)

2.i) How long was the same mask worn in average: 1-4 hours, during the whole duty shift, >1 day?

2.j) Were the employees trained in using the personal protective equipment?

3. Management of the pandemic

3.a) Were infectious disease specialists taking care of the personal protective equipment and trainings during the entire time of the pandemic?

3.b) How many infectious disease specialists were present during the pandemic in your institution?

3.c) Was a special task force in place for the management of the COVID-19 outbreak?

3.d) Was the hospital divided into zones to separate COVID-19 from non-COVID-19 patients?

3.e) Were COVID-19 positive patients cohorted in the hospital (i.e. more than one COVID-19 patient in one room)?

3.f) Was it mandatory for the health care workers to wear a mask in the whole hospital area?

3.g) Were hygiene and precaution trainings available for the health care workers (e.g. hand hygiene, how to dress the protective equipment)?

3.h) Were there any restrictions for visitors/relatives of the patients to enter the hospital and visit a patient?

4. Infection data

4.a) How many hospital beds are available in your institution?

4.b) How many employees work in your institution?

4.c) How many employees were infected with SARS-CoV2 in your institution?

4.d) How many COVID-19 patients were treated in your institution?

5. Would you like to make further comments?

German version

1. Vorbereitungen **vor dem COVID-19 Ausbruch**

1.a) Gab es Vorbereitungen oder Schulungen für eine Pandemie einer Atemwegsinfektion vor dem COVID-19 Ausbruch?

2. Schutzausrüstung **während des COVID-19 Ausbruchs im Falle von COVID-19 Patientenkontakt**

2.a) Welche Atemmasken wurden während des COVID-19 Ausbruchs benutzt: FFP2, FFP3, N95, KN95, oder chirurgische Masken? Andere Masken (z. B. Stoffmasken)? Keine Masken?

2.b) Wurden Einweghandschuhe verwendet?

2.c) Wurden Einwegkittel verwendet?

2.d) Wurden Einweghauben verwendet?

2.e) Wurden Einwegschutzbrillen verwendet?

2.f) Wurde anderes / zusätzliches Schutzmaterial verwendet?

2.g) War Händedesinfektionsmittel verfügbar?

2.h) Gab es einen Mangel an Schutzmaterial oder Händedesinfektionsmittel? Wenn ja, welches Material? (Masken? Handschuhe? Kleider? Mützen? Schutzbrillen? Händedesinfektionsmittel?)

2.i) Wie lange wurde dieselbe Maske durchschnittlich getragen: 1-4 Stunden während der gesamten Dienstschicht> 1 Tag?

2.j) Wurden die Mitarbeiter im Umgang mit der persönlichen Schutzausrüstung geschult?

3. Management der Pandemie

3.a) Haben sich Infektiologen während der gesamten Zeit der Pandemie um die persönliche Schutzausrüstung und die Schulungen gekümmert?

3.b) Wie viele Infektiologen waren während der Pandemie in Ihrer Einrichtung anwesend?

3.c) Wurde eine spezielle Task Force für die Bewältigung des COVID-19-Ausbruchs eingerichtet?

3.d) Wurde das Krankenhaus in Zonen unterteilt, um COVID-19 von Nicht-COVID-19-Patienten zu trennen?

3.e) Wurden COVID-19-positive Patienten im Krankenhaus gruppiert (d. h. mehr als ein COVID-19-Patient pro Raum)?

3.f) War es für das Gesundheitspersonal obligatorisch, im gesamten Krankenhausbereich eine Maske zu tragen?

3.g) Gab es Hygiene- und Vorsorgeschulungen für das Gesundheitspersonal (z. B. Händehygiene, Anziehen der Schutzausrüstung)?

3.h) Gab es Einschränkungen für Besucher / Angehörige der Patienten, das Krankenhaus zu betreten und einen Patienten zu besuchen?

4. Infektionsdaten

4.a) Wie viele Krankenhausbetten sind in Ihrem Krankenhaus verfügbar?

4.b) Wie viele Mitarbeiter arbeiten in Ihrem Krankenhaus?

4.c) Wie viele Mitarbeiter waren in Ihrem Krankenhaus mit SARS-CoV2 infiziert?

4.d) Wie viele COVID-19-Patienten wurden in Ihrem Krankenhaus behandelt?

5. Haben Sie noch weitere Anmerkungen?
